# Effects of Water Removal Devices on Ambient Inorganic Air Pollutant Measurements

**DOI:** 10.3390/ijerph16183446

**Published:** 2019-09-17

**Authors:** Dong-June Kim, Trieu-Vuong Dinh, Joo-Yeon Lee, In-Young Choi, Dong-Jin Son, In-Young Kim, Young Sunwoo, Jo-Chun Kim

**Affiliations:** Department of Civil and Environmental Engineering, Konkuk University, Seoul 05029, Korea, dinhtrieuvuong@gmail.com (T.-V.D.); joooyeon07@gmail.com (J.-Y.L.); force@konkuk.ac.kr (I.-Y.C.); kcalstyner@naver.com(D.-J.S.); dk1553@naver.com (I.-Y.K.); ysunwoo@konkuk.ac.kr (Y.S.)

**Keywords:** KPASS, cooler, ozone, sulfur dioxide, carbon monoxide, water removal device, ambient air pollutants

## Abstract

Water vapor is a pivotal obstacle when measuring ambient air pollutants. The effects of water vapor removal devices which are called KPASS (Key-compound PASSer) and Cooler. On the measurement of O_3_, SO_2_, and CO at ambient levels were investigated. Concentrations of O_3_, SO_2_, and CO were 100 ppb, 150 ppb, and 25 ppm, respectively. The amount of water vapor varied at different relative humidity levels of 30%, 50%, and 80% when the temperature was 25 °C and the pressure was 1 atm. Water vapor removal efficiencies and recovery rates of target gases were also determined. The KPASS showed a better performance than the Cooler device, removing 93.6% of water vapor and the Cooler removing 59.2%. In terms of recovery, the KPASS showed a better recovery of target gases than the Cooler. Consequently, it is suggested that the KPASS should be an alternative way to remove water vapor when measuring O_3_, SO_2_, and CO.

## 1. Introduction

As air pollution standards have been strengthened, technologies that measure air pollutants have continued to be developed [1]. However, air pollutants are difficult to measure because they are present in very small quantities (i.e., at levels of ppt to ppm) in the ambient air [2]. The main interference material when measuring ambient air pollutants is water vapor due to its extremely high concentration [3]. Water vapor can affect both negative and positive signals in the measurement of inorganic air pollutants, such as CO_2_ and NOx, depending on the measurement method [4,5]. In addition, several other problems can occur, such as a change in the retention time or baseline drift when measuring volatile organic compounds (VOCs) [6].

To eliminate these problems, a water removal device (WRD) is recommended [7]. According to standard analytical methods of air pollutants, WRDs should be employed when measuring SO_2_ and CO in the ambient air. For O_3_ analysis, the device should be calibrated with respect to the humidity level [8]. However, analyte loss in the water vapor removal process has been reported [3,5,7,9,10,11,12,13,14,15,16,17,18,19]. It was reported that thiols were lost up to 32% after removing humidity in a sample by a Nafion dryer [7]. Palluau et al. reported that ethylene, acetylene, propene, 1-butene, 1,3-butadiene, 1-pentene, 1-hexene, benzene, toluene, ethylbenzene and m/p-xylene were lost from 60 to 70% when a Nafion dryer was applied [6]. Methyl ethyl ketone and methyl isobutyl ketone were found to be lost approximately 13% when a cooler was used to remove water vapor [16]. In the water vapor removal process, a sample gas with high polarity and solubility might react with or be absorbed by the condensed liquid water [12]. In particular, analytes with high solubility and large dipole moments were influenced by water vapor, with the scale of influence depending on the measurement method [4,8,20,21,22,23,24]. Since an exact water removal process has not yet been specified, a clear and specific method is necessary. In addition, many problems with WRDs, such as low water vapor removal and low recovery of measured materials, have been reported [20,25]. Thus, the proper selection of a WRD is a pivotal issue.

In this study, we compared the performance of WRDs and investigated a suitable water removal method for the analysis of O_3_, SO_2_, and CO. 

## 2. Materials and Methods 

### 2.1. Experimental Apparatus

An O_3_ analyzer (ANA 4, Winstech Co., Ltd., Gunpo, Republic of Korea) was used in this study. The measurement method was UV Photometry. The analytical range of the analyzer was 0~500 ppb and the lower detection limit of the analyzer was less than 0.5 ppb. A fluorescence analyzer (43i, Thermo Fisher Scientific INC., Waltham, MA, USA) was used to analyze SO_2_, which had a lower detection limit of less than 1 ppb (Table 1). 

As a reference compound, an experiment measuring CO was conducted. A CO analyzer (Serinus 30, Ecotech Pty Ltd., Melbourne, Australia) was used for the CO analysis. The lower detection limit was 0.05 ppm. Table 1 describes the gas analyzer’s specifications for each target gas.

All analyzers except for ozone were calibrated with standard gases. The ozone analyzer was done with another approved analyzer (Korea Research Institute of Standards and Science, Daejeon, Republic of Korea). Two humidity sensors (Testo-645, Testo Ltd., Lenzkirch, Germany) were applied to measure humidity in this experiment. A KPASS (Key-compound PASSer)(MSD-P100, Nara Controls Inc., Seoul, Republic of Korea), which is an upgraded version of a Desolvator [26], was used as a WRD. The KPASS transformed water vapor into frost using a super-cooling phenomenon. A Cooler (SEC-2001B, Saehan high tech Co., Ltd., Ulsan, Republic of Korea) was also used as WRD to compare the removal of water vapor in the sample gases.

### 2.2. Materials

Zero air (99.99%, DongA Ltd., Anseong, Republic of Korea) was used to dilute the target gases. The O_3_ standard was produced by an ozone generator (DA-6200 Ozone generator, DongAn Information Industrial Co., Ltd., Daejeon, Republic of Korea). Both the O_3_ generator and the O_3_ analyzer were calibrated with other approved technologies (Korea Research Institute of Standards and Science, Republic of Korea). SO_2_ (10 ppm, Rigas Co., Ltd., Daejeon, Republic of Korea) and CO (100 ppm, Rigas Co., Ltd., Daejeon, Republic of Korea) were used for calibration and measurement.

### 2.3. Experimental Procedure

**Figure 1:**
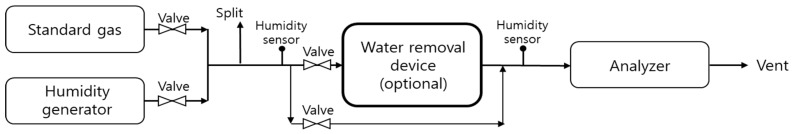
Experimental set-up for water vapor removal and target gas recovery.

Various concentrations (i.e., relative humidity) of water vapor were introduced into the WRDs to evaluate their performances. Each experiment was repeated three times. First, the experimental system was stabilized for 30 min. Then, the measurement data were collected every 5 min, and the data acquisition time interval was 5 s. The experimental set-up used is presented in Figure 1.

Humidity sensors were placed before and after the WRDs to observe water vapor removal (Figure 1). Control valves were used to change the experimental conditions. As shown in Table 2, the average relative humidity range was 45~68%, however, the maximum average relative humidity level was approximately 80%. We varied humidity as 0, 30, 50 and 80%. Hence, a water vapor generator was used to produce 2 L/min of humid air at 0%, 30%, 50%, and 80% RH. Furthermore, the measurement of ambient air pollutants was usually conducted at the air-conditioned monitoring site. Therefore, the laboratory temperature was maintained at 25 °C ± 1 °C. The sampling flow rate of the analyzer was 1 L/min. Zero air purging was done for 30 min after every experiment to maintain the same experimental conditions.

For recovery of the target gases, the standard gas was mixed with humid air to produce a sample gas with a consistent 1 L/min of flow rate. The target concentrations of O_3_, SO_2_, and CO were 100 ppb, 150 ppb, and 25 ppm, respectively. Dry samples, which composed with only zero air and standard gas, were also produced with the same concentrations to investigate the effect of humidity on the analyzers. Then, a paired t-test was conducted to compare concentrations of target gases obtained from their analyzers with respect to dry and humid conditions. Statistical analysis was conducted using Predictive Analytics Software (PASW 18, SPSS Inc, Hong Kong, China). The concentration of sample gases after penetrating WRDs were determined and compared with their initial concentrations. A paired t-test was also implemented for these comparisons.

## 3. Results

### 3.1. Humidity Removal

**Figure 2:**
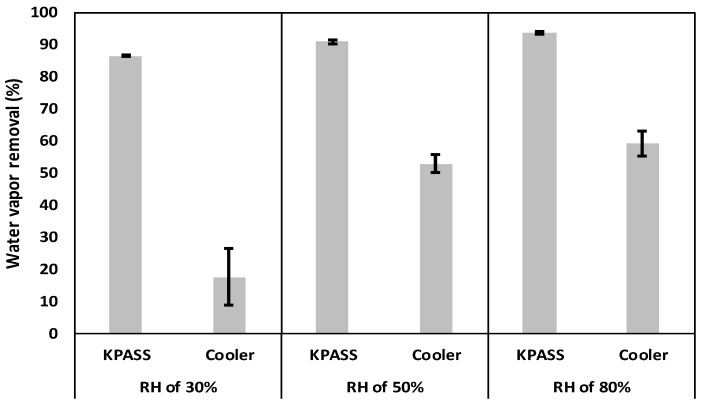
Water vapor removal using a KPASS (Key-compound PASSer) and a Cooler.

The performances of the KPASS and the Cooler are illustrated in Figure 2. As shown in Figure 2, at 30% RH, the KPASS and the Cooler removed 86.4% and 17.6% of water vapor, respectively. At 50% RH, the KPASS and the Cooler showed 90.8% and 52.7% water vapor removal, respectively. At 80% RH, 93.6% (KPASS) and 59.2% (Cooler) of water vapor removal were observed. The lowest water vapor removal was found in both devices when relative humidity (RH) was 30%. The performances of these devices improved with increasing RH. Since the outlet humidity of both water vapor removal devices was consistent, the lower inlet humidity indicated that less water removal occurred. Moreover, water removal by the Cooler was less than the KPASS at all humidity conditions, particularly under 30% RH where water removal was low. Conversely, both WRDs removed the most water at the high relative humidity condition of 80%.

The surface area of cooling of the KPASS was about 85 cm^2^, while the surface area of the Cooler was 220 cm^2^. In spite of that, the KPASS showed a higher water removal efficiency under all tested humidity conditions (Figure 2). Furthermore, the relative standard deviation (RSD) with respect to the removal efficiency of the KPASS was less than 0.7%. This inferred that the performance of the KPASS was consistent. There was a lack of comparison between the KPASS and the Cooler, but the KPASS was reported to have a higher and more stable water removal rate than a Nafion dryer [26]. A study on the water removal method using Nafion reported that recovery of the Nafion membrane to the polar material or the adsorbing material was inferior [28]. With insufficient heating, analytes absorbed and reacted with condensed water in the Nafion membrane [12,13,28]. The Nafion membrane was reported to have the same role as a Cooler due to insufficient heating or incomplete purging [12,29].

### 3.2. Humidity Impeding O_3_ Measurement

**Figure 3:**
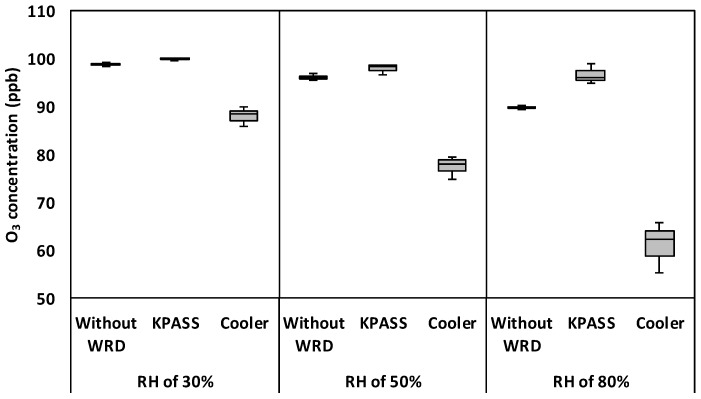
Box plot of O_3_ concentration with respect to water removal devices (WRDs) and RH conditions.

O_3_ at 100 ppb was used to investigate the recovery of WRDs. The experimental results are presented in Figure 3. 

As shown in Figure 3, O_3_ concentrations in the humid samples were 98.8 ppb, 96.2 ppb, and 89.7 ppb at RHs of 30%, 50%, and 80%, respectively. It was found that the mean of O_3_ concentration was statistically significant, different to mean initial O_3_ concentrations (*p*-value < 0.05). The decrement of O_3_ measurements caused by water vapor was also observed in other studies [4,6,9,14,25]. This was caused by uneven UV light through the detection cell and window if humidity existed. In this study, there was a loss of up to 11% of O_3_ concentrations in Figure 3, as also reported in another study [11]. 

When the KPASS was applied to remove water vapor in the sample gas, it was found that O_3_ concentrations were the same as its initial values (*p*-values > 0.05). This indicated that the KPASS did not affect O_3_ measurement [23]. In terms of the Cooler, the ozone concentrations were 88.0, 77.5, and 61.1 ppb. There was a statistically significant difference between mean initial O_3_ concentrations and those after penetrating the Cooler (*p*-values < 0.05). Hence, O_3_ was lost in the Cooler. According to Table A1 in Appendix A, O_3_ has a solubility of 1.885 × 10^−6^ and a dipole moment of 0.534, which is moderate compared to SO_2_ and CO. It was presumed that ozone is dissolved by and adhered to the water droplet in the Cooler due to the solubility and polarity of ozone. However, in the case of the KPASS, the influence of solubility and polarity on the O_3_ removal was negligible compared to those of the Cooler since water vapor was transformed into frost which is not a liquid but solid material. Accordingly, KPASS could remove water vapor in sample flow without any influence on O_3_.

### 3.3. SO_2_ Analysis Hindered by Humidity

**Figure 4:**
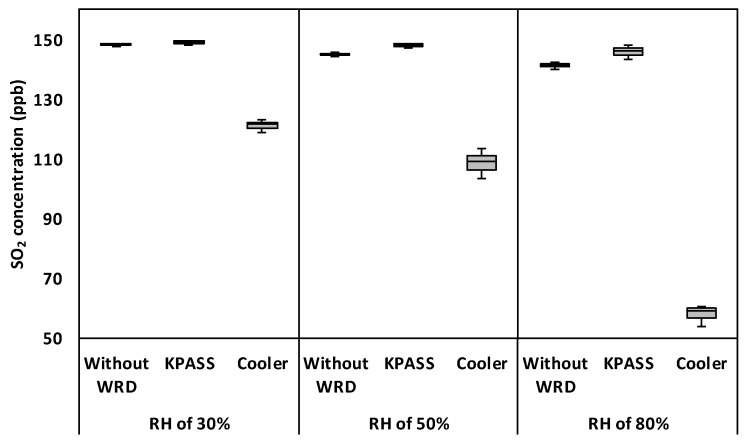
Box plot of SO_2_ concentration with respect to WRDs and RH conditions.

The initial concentration of SO_2_ was 150 ppb. As shown in Figure 4, SO_2_ concentrations in the humid samples were 147.9 ppb, 144.6 ppb, and 139.8 ppb at 30% RH, 50% RH, and 80% RH, respectively. It was found that the mean of SO_2_ concentration in the humid sample had a statistically significant difference to mean initial SO_2_ concentrations (*p*-value < 0.05). This was attributed to the quenching effect of water vapor during the SO_2_ measurement [10]. The quenching effect caused by the humidity may have led to underestimation of the SO_2_ concentration [7].

When the KPASS was employed to remove the water vapor in the samples, SO_2_ concentrations obtained from the SO_2_ analyzer were same with its initial concentration (*p*-values > 0.05), except the case of 50% RH. Although KPASS’s *p*-value was lower than 0.05 in the case of 50% RH, it showed high recovery (i.e., 98.6%). In terms of the Cooler, SO_2_ recovery rate declined as humidity increased (*p*-values < 0.05). At 30% RH, SO_2_ concentrations of 149 ppb and 121 ppb were observed after passing through the KPASS and the Cooler, respectively. At 50% RH, SO_2_ concentrations were 148 ppb and 108.6 ppb, respectively. At 80% RH, SO_2_ concentrations were 145.8 ppb and 57.8 ppb, respectively. On the other hand, RSD results were less than 5% for both the KPASS and the Cooler. At relative humidity of 30% and 80%, the KPASS showed better performance than the Cooler based on t-test results. In case of relative humidity of 50%, SO_2_ concentration after passing through the KPASS was statistically significantly different from its initial concentration (*p*-value = 0.038). However, actual SO_2_ recovery rate for the KPASS was 98.6 %. In contrast, with respect to the Cooler, was relatively low 72.4% at the same condition. Therefore, it might be concluded that KPASS’s performance was better than that of Cooler’s in terms of SO_2_ measurement.

The SO_2_ showed a higher level of water solubility and a larger dipole moment than CO and O_3_ (see Table A1) [30]. Due to its high solubility and large dipole moment, SO_2_ was considered to be removed because it was dissolved by and adhered to water droplet [12]. The SO_2_ was significantly influenced by WRDs like Coolers and Nafion dryers [12]. The KPASS showed better recovery rate than that of the Cooler. The different phases of water in the KPASS and the Cooler brought about this recovery pattern. The Cooler removed the water vapor in the form of liquid droplets, thus, SO_2_ was easily absorbed by the water droplets due to its higher solubility and dipole moment. This phenomenon suggests that Coolers should not be suitable for SO_2_ measurement. 

### 3.4. CO Measurement with Humidity

CO has the smallest dipole moment and least solubility in water according to Table A1. Therefore, the experiment was conducted by selecting CO as the reference gas [31]. The CO sample gas was generated by mixing CO standard gas with zero air. The CO standard gas concentration was 25 ppm. Without WRD, initial CO concentrations were observed as 25.1 ppm, 24.7 ppm, and 24.8 ppm, respectively, at RH values of 30%, 50%, and 80%. It was found that there was no statistically different among initial CO concentrations (*p*-values > 0.05) according to the humidity change.

**Figure 5:**
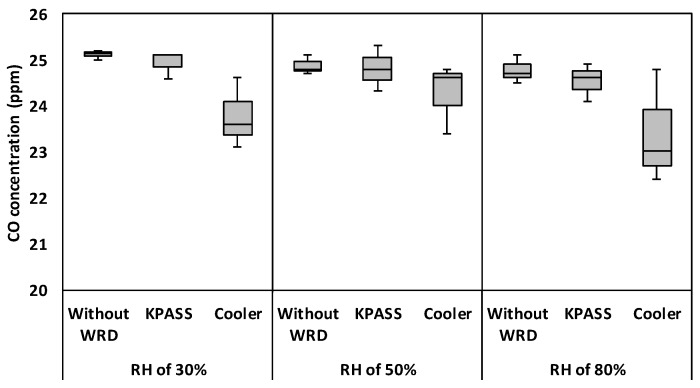
Box plot of CO concentration with respect to WRDs and RH conditions.

After passing through the WRD, CO concentrations were measured at 24.9 ppm and 23.8 ppm for the KPASS and Cooler devices at 30%RH, respectively. Likewise, they were 24.8 ppm and 24.3 ppm at 50% RH, and 24.5 ppm and 23.4 ppm at 80% RH, respectively. It indicated that all CO concentrations after using WRD were the same as their initial concentrations (*p*-values > 0.05). As shown in Figure 5, CO concentrations with respect to the Cooler were more unstable than those to the KPASS in all the humidity of concern although statistical analysis showed no significant difference with both WRDs (*p*-values > 0.05). In the other study, it was reported that CO measurement using a non-dispersive infrared analyzer interfered with water vapor [15]. In short, the influence of water vapor on the CO measurement was negligible compared to those of other compounds (O_3_, SO_2_) since its solubility and polarity are lower than others. Accordingly, any type of WRDs can be applied with no influence on the CO measurement.

## 4. Conclusions

Water removal and the effect of the WRD (i.e., KPASS and Cooler) on the measurement of ozone, sulfur dioxide, and carbon monoxide were evaluated in this study. The removal of water vapor and the recovery rate of target gases were observed. It was found that the water removal of the KPASS was approximately 60% higher than that of the Cooler. In terms of the recovery rate of target analytes, the Cooler showed lower recovery rates than the KPASS in all cases of concern. In case of O_3_, its recovery rates associated with the KPASS were approximately 35% higher than those with the Cooler. In terms of SO_2_, the KPASS showed approximately 60% higher recovery rate than the Cooler. Furthermore, high RSD values were observed with respect to the Cooler. This demonstrated that using the KPASS resulted in the consistent measurement for target gases. Moreover, it was found that SO_2_ and O_3_, which have large dipole moments and high solubility, were affected by the Cooler more than by the KPASS, whereas it suggested that the KPASS helped to improve the accuracy of measurements. 

In general, advantages of the KPASS are higher water removal efficiencies and recovery rates of analytes. However, the initial cost of the KPASS might be higher than that of the Cooler. Therefore, we consider the KPASS to be a suitable WRD with respect to inorganic air pollutant measurements under the condition of this study. If the KPASS is applied for the O_3_ and SO_2_ measurement, the accuracy of inventory data could be improved. This might improve O_3_ assessment and forecasting in the ambient air. That is a significant engineering of the KPASS in the environmental field. There is a need for further studies regarding various WRDs and analytes.

## Figures and Tables

**Table 1:** Specifications of target gas analyzer.

Model Name	ANA 4 O_3_ Analyzer	43i SO_2_ Analyzer	Serinus 30 CO Analyzer
Target gas	Ozone	Sulfur dioxide	Carbon monoxide
Range	0–500 ppb	−10,000 ppb	0–200 ppm
Lower detection limit	0.5 ppb	1 ppb	0.05 ppm
Linearity	± 1% of span gas concentration	± 1% of full scale	± 1% of full scale
Zero drift	< 1 ppb	< 1 ppb	< 0.1 ppm
Span drift	± 1% of full scale per day	± 1% of full scale per week	0.5% of reading per day

**Table 2:** Climate data from Seoul in 2018.

Month	Average Temperature (°C)	Average Water Vapor Pressure (hPa)	Average Relative Humidity (%)	Ref.
Jan-18	−4	2.5	48	[27]
Feb-18	−1.6	2.6	45	[27]
Mar-18	8.1	6.6	59	[27]
Apr-18	13	8	55	[27]
May-18	18.2	13.1	63	[27]
Jun-18	23.1	17.3	63	[27]
Jul-18	27.8	24.6	68	[27]
Aug-18	28.8	24.9	65	[27]
Sep-18	21.5	15.4	61	[27]
Oct-18	13.1	8.9	59	[27]
Nov-18	7.8	6.3	58	[27]
Dec-18	−0.6	3.1	46	[27]

## Appendix A

**Table A1:** The Dipole moment and solubility of molecules in the gas phase.

Molecule	Dipole Moment	Solubility (Mole Fraction)
O_3_	0.534	1.885 × 10^−6^
SO_2_	1.633	2.90 × 10^−2^
CO	0.110	1.918 × 10^−5^
H_2_O	1.854	-

Note: dipole moment is given in debye unit (D = 3.33564 × 10^−30^ cm).
